# Peripersonal Visuospatial Abilities in Williams Syndrome Analyzed by a Table Radial Arm Maze Task

**DOI:** 10.3389/fnhum.2020.00254

**Published:** 2020-07-20

**Authors:** Francesca Foti, Pierpaolo Sorrentino, Deny Menghini, Simone Montuori, Matteo Pesoli, Patrizia Turriziani, Stefano Vicari, Laura Petrosini, Laura Mandolesi

**Affiliations:** ^1^Department of Medical and Surgical Sciences, Magna Graecia University of Catanzaro, Catanzaro, Italy; ^2^Department of Engineering, Parthenope University of Naples, Naples, Italy; ^3^Child Neuropsychiatry Unit, Neuroscience Department, “Children’s Hospital Bambino Gesù”, Rome, Italy; ^4^Department of Movement Sciences and Wellbeing, Parthenope University of Naples, Naples, Italy; ^5^Department of Psychology, Educational Sciences and Human Movement, University of Palermo, Palermo, Italy; ^6^Child and Adolescent Neuropsychiatry Unit, Department of Neuroscience, Bambino Gesù Children’s Hospital (IRCCS), Rome, Italy; ^7^Department of Life Sciences and Public Health, Catholic University, Rome, Italy; ^8^Laboratory of Experimental and Behavioural Neurophysiology, IRCCS Fondazione Santa Lucia, Rome, Italy; ^9^Department of Humanistic Studies, University of Naples Federico II, Naples, Italy

**Keywords:** spatial exploration, spatial memory, ecological behavioral task, children, navigation abilities

## Abstract

Williams syndrome (WS) is a genetic deletion syndrome characterized by severe visuospatial deficits affecting spatial exploration and navigation abilities in extra-personal space.To date, little is known about spatial elaboration and reaching abilities in the peripersonal space in individuals with WS. The present study is aimed at evaluating the visuospatial abilities in individuals with WS and comparing their performances with those of mental age-matched typically developing (TD) children by using a highly sensitive ecological version of the Radial Arm Maze (table RAM). We evaluated 15 individuals with WS and 15 TD children in two different table RAM paradigms: the free-choice paradigm, mainly to analyze the aspects linked to procedural and memory components, and the forced-choice paradigm, to disentangle the components linked to spatial working memory from the procedural ones.Data show that individuals with WS made significantly more working memory errors as compared with TD children, thus evidencing a marked deficit in resolving the task when the mnesic load increased. Our findings provide new insights on the cognitive profile of WS.

## Introduction

Williams syndrome (WS) is a rare genetic disorder, with a prevalence of 1 in 7,500–1 in 20,000 (Stromme et al., 2002), without gender preference, caused by a microdeletion on chromosome 7q11.23 (Ewart et al., 1993; Koehler et al., 2014).

WS has drawn the attention of cognitive neuroscientists as a result of an uneven cognitive profile with selective weak points in visuospatial abilities, and relative strength points in verbal abilities and face recognition (Atkinson et al., 2001; Bellugi and St George, 2001; Vicari et al., 2001; Searcy et al., 2004). In relation to their visuospatial deficit, WS individuals fail selectively on tasks requiring to decipher, judge, recall, and reconstruct the relationship between forms and objects (e.g., draw a house, replicate a block design, recall where an object was previously seen on a page, determine the orientation of a line; Bellugi et al., 1999; Mervis et al., 2000; Vicari et al., 2005; Martens et al., 2008; Farran et al., 2013; Broadbent et al., 2014a; Farran and Dodd, 2015).

In the last decade, behavioral studies based on large-scale spatial tasks elucidated how the visuospatial deficits of WS individuals influence exploration of large environments (Mandolesi et al., 2009a; Smith et al., 2009; Farran et al., 2010, 2012; Farran and Dodd, 2015; Farran et al., 2019; Foti et al., 2011; Broadbent et al., 2014b, 2015).

Importantly, the exploration and orientation in new and known environment represent spatial abilities needed in everyday life and, therefore, a prerequisite to autonomy and social integration. In the same way, it is important to be able to explore, know, and understand how peripersonal space is organized. These abilities allow to interact with objects by correctly interpreting what they are for. Hence, the study of spatial exploration proves relevant for the implementation of intervention programs in cognitive disabilities in general, and in WS in particular.

To explore an environment, the subject has to gain knowledge of the position of the environmental cues and of his/her own position with respect to these (O’Keefe and Nadel, 1978; Stupien et al., 2003). This type of spatial knowledge is referred as “declarative” (Jarrard, 1993). In wayfinding and navigation task, the declarative knowledge is mainly related to egocentric and allocentric encoding. Egocentric coordinates refer to the positions of environmental cues with respect to the subject, while allocentric coordinates represent the position of a cue in relation to another cue and then irrespective of the position of the subject (Arleo and Rondi-Reig, 2007).

On the other hand, the subject needs to understand how to move in the environment to reach (or avoid) specific cues (Mandolesi et al., 2009b). This type of knowledge is referred to as “procedural” (Foti et al., 2011) and in wayfinding and navigational tasks the procedural knowledge is highly correlated to exploration strategies.

It is well accepted that declarative and procedural spatial abilities are equally necessary for an efficient exploration and for the construction of a spatial map (O’Keefe and Nadel, 1978; Mandolesi et al., 2009b). Furthermore, these processes are strongly correlated with spatial working memory (Sorrentino et al., 2019). In conclusion, given its complex and multi-faceted nature, the study of spatial exploration requires taking into account all the spatial abilities involved.

Many researchers investigating spatial exploration in WS have mainly focused on the different facets of declarative knowledge, analyzing egocentric and allocentric encoding by means of wayfinding and navigational tasks (Bernardino et al., 2013). From these studies, it emerges that individuals with WS have difficulty to estimate the relation between landmarks and specific items within an environment (Farran et al., 2010; Broadbent et al., 2014a), and to employ a sequential egocentric strategy to guide the learning and retracing of a route (Broadbent et al., 2015). This evidence suggests a deficit in allocentric and egocentric encoding that could be explained by anatomical and functional alterations in the hippocampus (Meyer-Lindenberg et al., 2005), and by the well-documented deficit in the dorsal stream (Atkinson et al., 2001; Meyer-Lindenberg et al., 2005)—the neural pattern related to the link between perception and action.

Along with behavioral studies on WS, our research group mainly focused on the study of explorative abilities of WS individuals by means of two highly ecological walking spatial tasks: the Radial Arm Maze (RAM) and the Open Field with multiple rewards (OFmr). Both spatial tasks revealed the presence of procedural and memory deficits in WS (Mandolesi et al., 2009a; Foti et al., 2011).

While exploration abilities in WS have been extensively studied with the large-scale tasks, little is known about the exploration of peripersonal space in this syndrome. This space has a key functional role, as it is where all physical interactions with objects in the environment occur (Serino, 2019). The binding of visual information arising outside the body with tactile information arising on the body allows the representation of the space lying in between. This peripersonal space is often the theater of interactions with objects, supports self-location, contributes to bodily self-consciousness, and mediates higher-level cognitive functions (Serino, 2019).

The objective of the present study is to study whether the deficit in visuospatial information processing in the extra-personal space evidenced in WS individuals was also present during the exploration of the peripersonal space. Hence, in the present research, we evaluated the peripersonal visuospatial abilities in individuals with WS by using a table version of the Radial Arm Maze task (table RAM) and compared their performances with those of mental age-matched typically developing (TD) children.

## Materials and Methods

### Participants

Fifteen individuals with WS (nine males and six females) and 15 mental age- and gender-matched TD children were recruited to participate in the study. All WS individuals (mean chronological age, 18.1 years ± 5.2) and TD children (mean chronological age, 6.5 years ± 0.5) were right-handed and native Italian speakers. Participants with WS were recruited at the Children’s Hospital Bambino Gesù in Rome and at WS Association Marche in Fano (PU, Italy). Clinical diagnosis of WS was confirmed by genetic investigation (fluorescence *in situ* hybridization) demonstrating the deletion on the chromosome band 7q11.23. All participants live home with their families in Italy. The participants’ cognitive level was measured using the short version of the Leiter-R intelligence scale (Roid and Miller, 2002). Mean mental age in the WS group was 6.2 years ± 0.8 and in the TD group was 6.5 years ± 0.6, whereas mean intelligence quotient (IQ) was 55.7 ± 7.3 and 103.5 ± 6.0, respectively. Overall, the groups differed in chronological age (*F*_(1,28)_ = 73.9, *p* < 0.00001, $\eta_{\text{p}}^{2}$ = 0.73) and IQ (*F*_(1,28)_ = 386.4, *p* < 0.00001, $\eta_{\text{p}}^{2}$ = 0.93), but not mental age (*F*_(1,28)_ = 1.6, *p* = 0.22, $\eta_{\text{p}}^{2}$ = 0.054).

Written informed consent was obtained from all the parents of the participants. The study had been approved by the Ethics Committee of Bambino Gesù Children’s Hospital, Rome, Italy (protocol number 486 LB) and was carried out in accordance with the declaration of Helsinki.

### Table Radial Arm Maze (table RAM)

**Figure 1:**
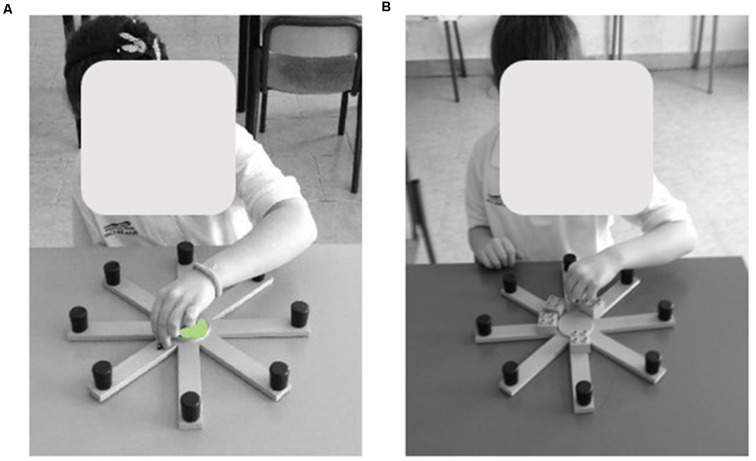
Views of the table Radial Arm Maze (RAM). The figure shows two typically developing (TD) children performing the “Ladybug game” by means of table RAM in the free-choice **(A)** and forced-choice **(B)** paradigms of the task. Written informed consent was obtained from the parents of children for the publication of this image.

The table RAM is made of a round central platform (5 cm in diameter). Eight green arms (3 cm wide × 25 cm long) depart the central platform like the spokes of a wheel (Figure 1). At the end of each arm, a small black round cap (1 cm in diameter × 2 cm height) covered the reward (a little colored wooden ladybug).

The table RAM was placed on a desk while the extra-maze cues (windows, paintings, posters, doors, and experimenter) were held in constant spatial relations throughout the experiment. The arms were virtually numbered clockwise, arm 1 being in front of the subject. Participants had visual access to the table RAM only during the experiment.

Although the participant is seated in front of the RAM, this task can be considered as a peripersonal visuospatial task because it forces the child to explore a portion of space accessible with the limbs.

### Experimental Procedure

The table RAM task was presented as “Ladybug game.” The child had to move the older sister ladybug (“Ladybug”), placed on the central platform, to find its sisters hidden inside the caps at the end of each arm.

To increase motivation, at the end of each trial the child received a reward (a coin) in exchange for the ladybugs discovered.

The children were evaluated in two different table RAM paradigms: the *free-choice paradigm*, to analyze the peripersonal procedural and memory components, and the *forced-choice paradigm*, to disentangle the components linked to spatial memory from the procedural ones. The choices made by the participants in each trial of both paradigms were videotaped and registered manually.

#### Free-Choice Paradigm

Each child could explore the eight arms freely to find the ladybugs hidden inside the caps at the ends of each arm. A trial was counted as successful when all eight ladybugs were collected. Afterwards, the child could keep the Ladybug for a short time to verify whether he/she was aware of having finished the trial. In other words, the experimenter observed whether the child revisited a previously explored arm to find a further ladybug in the upturned caps. After a supplementary incorrect visit, the child was informed that the game was over.

A trial ended when all eight ladybugs had been collected, when 20 choices had been made, or after 5 min from the start of the task. Since each cap contained one ladybug, the optimal performance entailed eight visits to the arms, that is, visiting each upturned cap only once.

If the subject visited the same arm twice during the same trial, it was considered as an error. All participants performed three trials in 1 day (one session). The inter-trial interval was at least 1 h long.

At the beginning of the first trial, the experimenter used the same simple verbal instructions to explain the task to each participant (“Now we will play a little game in which the Ladybug has to find its sisters hidden in these black upturned caps. On my mark, make the Ladybug move. Remember not to let it get out of the corridors, and always return it to the center. Then, put its sisters at the center so they won’t hide anymore. Go and enjoy yourself!”). Immediately after the instructions were given, the participants started the task and no further instructions and verbal encouragement were provided. All participants displayed no hesitation when starting the task. In fact, all participants did not spend more than 1–2 s before moving the Ladybug on the central platform. The trial was void if the child made the Ladybug exit the maze. All participants ended the trials in the required time. To assess the subjects’ performance in the free-choice paradigm, we evaluated the following parameters: *search efficiency*, defined as the percentage of correctly visited arms divided by the total number of visits; *the longest sequence of correctly visited arms* (the sequence ranged from 1 to 8); *error-free trials*, calculated as the number of trials without errors; *adjacent visits*, calculated as the percentage of visits in adjacent arms (i.e., visiting arm 3 and then arm 4) divided by the number of visits; *declarative mastery*, a binary index classifier of the participant’s behavior at the end of the trial, defined as 0 if they wrongly continued the search, and as 1 otherwise.

#### Forced-Choice Paradigm

The day after the session of the free-choice RAM paradigm, the participants were tested in the forced-choice paradigm of table RAM. In the first phase, although all arms contained the ladybugs on them, only four arms (for example, arms 1, 3, 4, 7) were accessible because the remaining four arms were closed by a Lego cube (2 cm × 2 cm × 2 cm) at the proximal end of each arm. Different angles separated the opened arms to avoid the subjects reaching the solution through the employment of a procedural strategy, such as for example performing only adjacent angles. The table RAM task started with the Ladybug placed on the central area of the maze and the participant was allowed to explore the four open arms by moving the Ladybug to collect the four accessible ladybugs. Afterwards, the participant was invited to interrupt the game and she/he was kept in a separate place without seeing the game and chatting with the experimenter for 120 s before the second phase of the task started. In the second phase, the participant was allowed moving the Ladybug in all arms, but only the four previously closed arms were rewarded (since the other four ladybugs had been collected in the previous phase). The successes in visiting only the rewarded arms essentially depended on remembering which arms had already been visited, stressing the memory component and neglecting the search patterns.

Each participant performed three trials a day for two consecutive days (two sessions), with an inter-trial interval of at least 1 h. In each of the six trials, a different configuration of closed arms was used. The arms opened in the first phase were never contiguous nor separated by regular patterns of angles (e.g., the opened arms never were 1, 2, 3, 4, or 1, 3, 5, 7).

At the beginning of the first trial, the experimenter explained the task to each participant using the same simple verbal instructions (“Do you remember the Ladybug that had to find its sisters under the black upturned caps? Well, the mischievous sisters have hidden themselves again. However, they do not know you’re there to help the Ladybug find them. Now, some corridors are blocked. You have to let the Ladybug enter only in the arms that are open. Go!”). The verbal instructions after the 120-s interval were: “Uh! Something now has changed, there are no Lego bricks anymore. Then, the Ladybird can freely go and look for the other sisters! Good job!”).

In the forced-choice paradigm, the parameter taken into account was the* short-term memory errors*, defined as the re-visits into already visited arms. This parameter was broken down further into two error subtypes: *across-phase errors*, defined as visits into an arm that had been visited during the first phase of the same trial; *within-phase errors*, defined as re-visits into an arm already visited in the same phase. We considered also *the longest sequence of correctly visited arms*. In this case, the sequence ranged from 1 to 4.

### Statistical Analyses

All data were presented as the mean ± SD and were first tested for normality (Shapiro–Wilk’s test) and homoscedasticity (Levene’s test). When normally distributed, data were analyzed by using one-way or two-way analyses of variance (ANOVAs). When data were not normally distributed, non-parametric analyses (Mann–Whitney *U*, Wilcoxon’s test) were used. The error-free trials and the declarative mastery were evaluated using *χ*^2^ metric. Analyses were performed by using Statistica 8.0; the significance level was defined as *p* < 0.05. Giving the numerous analyses, controlling for the alpha inflation was needed. We controlled the proportion of type I errors among all rejected null hypotheses by setting the false discovery rate (FDR) to 0.05. The FDR was estimated through the procedure described in Benjamini and Hochberg (1995). In our results, the 0.05 level of significance reported was pre- and post-FDR correction.

## Results

### Free-Choice Paradigm

#### Search Efficiency

**Figure 2:**
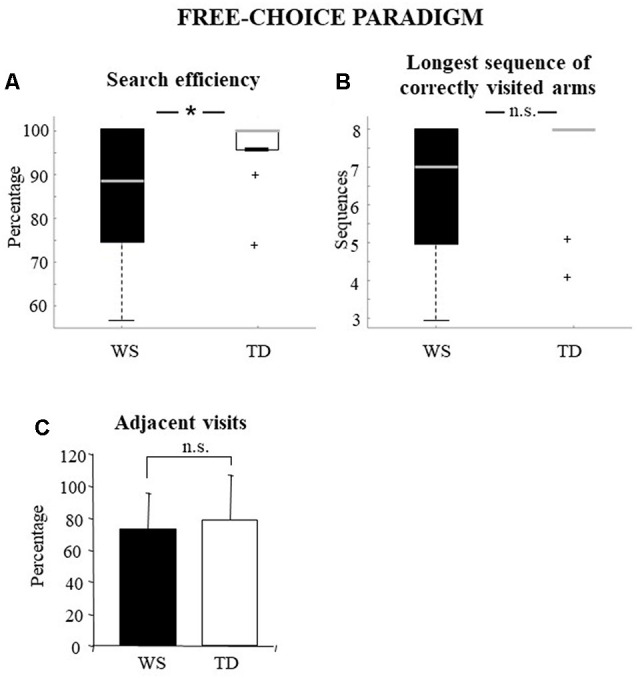
Performances of the Williams syndrome (WS) participants and TD children in free-choice paradigm. Data are expressed as median and quartiles **(A,B)** and mean ± SD **(C)**. The asterisk indicates the significance level of Mann–Whitney *U* (*p < 0.05), +indicates outliers. ns = no significant difference.

The percentage of correct visits represents a general parameter that indicates the efficiency of processing of peripersonal space. Therefore, it can be considered as a parameter that expresses all the others in their entirety. Individuals with WS obtained significantly lower values of search efficiency than TD children (WS: median = 88.89, *q*_1_ = 76.4, *q*_3_ = 100; TD: 100, *q*_1_ = 96, *q*_3_ = 100; Mann–Whitney *U* = 68.5, *Z* = −1.99, *p* = 0.04, *p*_FDR_ = 0.049; Figure 2A).

#### Error-Free Trials

This parameter more precisely reflects the search efficiency, that is, the degree of correctness of the task. When the error-free trials were considered, the performance observed in WS and TD groups was significantly different. Indeed, the WS participants performed a significantly lower number of error-free trials than TD participants (WS vs. TD: 6 vs. 12; *η*^2^ = 5; *df* = 1; *p* = 0.02, *p*_FDR_ = 0.027).

#### The Longest Sequence of Correctly Visited Arms

This parameter indicates the longest sequence of correctly visited arms. High values could be obtained by exploiting the working memory and mapping abilities or by efficient explorative strategies. The longest sequence of correctly visited arms of WS and TD groups was not significantly different (WS: median = 7; *q*_1_ = 5; *q*_3_ = 8; TD: 8; *q*_1_ = 7.2; *q*_3_ = 8; Mann–Whitney *U* = 78.5, *Z* = −1.53, *p* = 0.12, *p*_FDR_ = 0.132; Figure 2B).

#### Adjacent Visits

The exploration of adjacent arms indicates how well the procedural strategy of research is put into action. High percentages indicate that the task is solved without (or with little) mnesic load and is correlated to the parameter reporting the longest sequence of correctly visited arms. The percentages of adjacent visits of WS and TD groups showed no significant difference (WS: $\bar{x}$ = 72.7 ± 22.54; TD: $\bar{x}$: 79.1 ± 28.09; one-way ANOVA: *F*_(1, 28)_ = 0.42, *p* = 0.52, *p*_FDR_ = 0.520, $\eta_{\text{p}}^{2}$ = 0.01; Figure 2C).

#### Declarative Mastery

The awareness of having concluded the task represents a parameter correlated to the mapping abilities and/or to rules learned. A significantly higher proportion of WS individuals was not aware of having completed the task as compared to TD group (*η*^2^_(*df* = 1)_ = 2.16; *p* = 0.014, *p*_FDR_ = 0.022).

### Forced-Choice Paradigm

#### Short-Term Memory Errors

**Figure 3:**
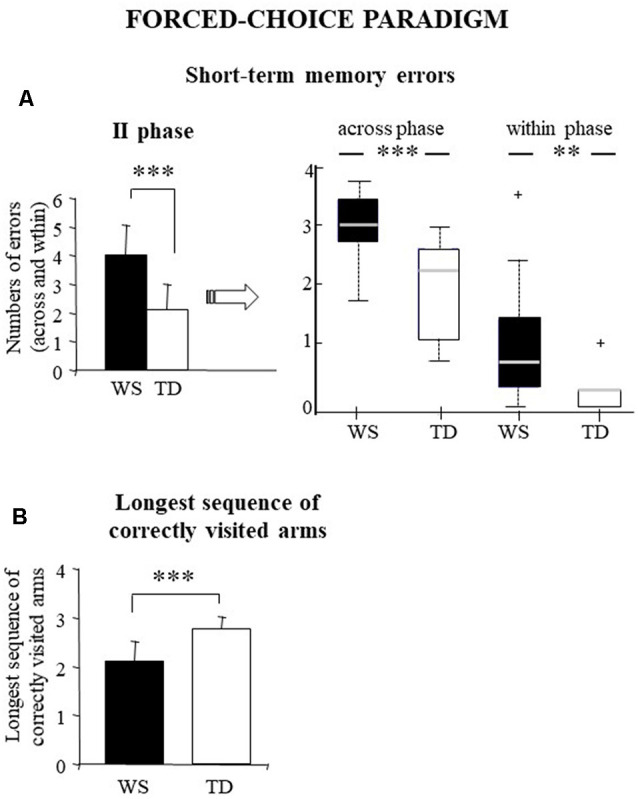
Performances of the WS and TD participants in forced-choice paradigm. Data are expressed as mean ± SD: (left side of part **A** and **B**) and median and quartiles (right side of part **A**). The asterisks indicate the significance level of ANOVAs and Wilcoxon’s test (**p < 0.005, ***p < 0.00005). ^+^indicates outliers.

This parameter as a whole indicates a possible deficit in short-term memory processes without specifying the kind of the memory deficit. A one-way ANOVA was run on short-term memory errors performed in the second phase of the test when all the arms were opened and the participants could move the Ladybug without restrictions. Statistical analysis revealed that WS individuals made a significantly higher number of short-term memory errors than TD children (WS: = 4 ± 1.08; TD: $\bar{x}$ = 2.1 ± 0.91; *F*_(1, 28)_ = 27.02, *p* = 0.00002, *p*_FDR_ = 0.00011, $\eta_{\text{p}}^{2}$ = 0.49; Figure 3A).

To better understand the kind of the memory deficit, we have analyzed the two subtypes of short-term memory errors: *across-phase errors* (visits into an arm that had been entered during the first phase) and *within-phase errors* (re-visits into an arm previously visited in the same phase). While across-phase errors reflect a short-term deficit, within-phase errors express a working memory deficit. Non-parametric analyses (Mann–Whitney) revealed that WS individuals made a significantly higher number of across-phase and within-phase errors compared with TD children (across-phase errors: *U* = 31, *Z* = 3.38, *p* = 0.0007, *p*_FDR_ = 0.0001; within-phase errors: *U* = 38, *Z* = 3.09, *p* = 0.002, *p*_FDR_ = 0.004). Moreover, non-parametric analyses (Wilcoxon’s test) revealed that both groups made more across-phase errors than within-phase (WS: *Z* = 3.29, *p* = 0.0001, *p*_FDR_ = 0.0004; TD: *Z* = 3.41 *p* = 0.0006, *p*_FDR_ = 0.002; Figure 3A).

#### The Longest Sequence of Correctly Visited Arms

In this paradigm, this parameter ranged from 1 to 4. High scores in this parameter express a correct functioning of working memory processes. In fact, to prevent solving the task by using procedural strategies, in the second phase the correct arms were never contiguous nor separated by regular patterns of angles. WS individuals obtained significantly lower scores of the longest sequence of correctly visited arms than TD children (WS: $\bar{x}$ = 2.1 ± 0.42; TD: $\bar{x}$: 2.8 ± 0.24; one-way ANOVA: *F*_(1,28)_ = 28.79, *p* = 0.00001, *p*_FDR_ = 0.0001, $\eta_{\text{p}}^{2}$ = 0.51; Figure 3B).

## Discussion

The present study focused on the analysis of the processing of the peripersonal space in individuals with WS by using a small-scale behavioral task that allows distinguishing memory components from procedural ones. In particular, we used a table version of the RAM that reproduces in small scale the classical walking RAM task (Overman et al., 1996; Mandolesi et al., 2009a). We tested WS individuals according to two RAM paradigms: the free-choice and forced-choice paradigms that allow to evaluate different facets (procedural and memory abilities) of the spatial function. In fact, while the free-choice paradigm allows to evaluate the possible presence of a global spatial deficit (without distinguishing procedural from memory abilities) and of impaired declarative abilities (by means of the declarative mastery parameter), the forced-choice paradigm allows to realize more precisely the nature (procedural or mnesic) of the deficit, providing also the distinction between short-term and working-memory errors.

The main result of the present research is the deficit of WS individuals in the peripersonal space based mainly on impaired memory abilities. Such a memory deficit emerged clearly analyzing the performances of the WS individuals in the forced-choice paradigm that singles out short-term from working memory abilities. In fact, WS individuals made significantly more within-phase errors and obtained lower values of the longest sequence of correctly visited arms (Figures 3A,B) in comparison with TD children.

A memory deficit was evident, although more mildly, even in the free-choice condition, when WS participants obtained a success rate lower than TD children (Figures 2A,B). Again, when procedural abilities were considered, the WS participants performed similarly to the TD children. In fact, the two groups of participants made a comparable number of visits in adjacent arms (Figure 2C), thus putting in place an efficient search strategy. The procedural strategy of visiting adjacent arms is captured by the longest sequence of correctly visited arms, a parameter that in this paradigm was similar in both groups (Figure 2B). It is important to stress that, despite the WS individuals used exploration strategies similar to the TD children, unlike them, the WS participants did not realize that the task was finished (as indicated by the declarative mastery parameter), once again confirming the presence of a deficit in visuospatial information processing.

The data described in both our studies with walking RAM task (Mandolesi et al., 2009a) and table RAM task highlight that in WS the deficit in the elaboration of the allocentric space is different from that in the peripersonal space. While in the elaboration of the peripersonal space the WS individuals exhibited memory but not procedural deficits, in the elaboration of the allocentric space they exhibited, beside the memory deficit, also remarkable procedural deficits (Mandolesi et al., 2009a; Farran et al., 2012, 2016; Foti et al., 2013; Broadbent et al., 2014b). Such diversity of processing of near and far space may be explained by taking into account the sensorimotor and cognitive processes recruited as well as the neuronal circuitry involved in the two conditions. In fact, walking tasks involving the exploration of extra-personal space (as walking RAM task) require to process the proprioceptive, vestibular, visual information derived from signals related to locomotory movements, as well as path integration processes in which the self-motion signals are processed in conjunction with external location-based references.

Conversely, tasks involving the processing of body–objects interaction in peripersonal space (as table RAM task) imply not only low-level sensorimotor representations of the space around the different body parts (in the present case, mainly upper limbs), but also the coding of multisensory signals in reference frames, to which visual and proprioceptive signals on the body parts location in space strongly contribute. Notably, it has been reported that tactile and visual stimuli inside the peripersonal space elicit a stronger processing and induce a more powerful multisensory activation than stimuli outside the peripersonal space (Serino, 2019).

The spatial memory deficit exhibited by WS participants in the table RAM task finds correspondence in the deficits of WS individuals when performing the Corsi Block task or block construction tasks (Vicari et al., 1996; Jarrold et al., 1999; Farran et al., 2001; Hoffman et al., 2003; Farran and Jarrold, 2005; Sampaio et al., 2008).

In addition to the different processing of the information linked to self-motion signals or to body–object interaction, it has to be taken into account that, while in the walking RAM task, the participants are inside the maze and see it from inside, in the table RAM task, the participants see the whole maze from above. The different view of the maze (from inside or from above) triggers different mental processes related to the construction of the spatial map. In the first case (vision from inside/RAM walking task), the participant is compelled to build a spatial cognitive map of RAM to orient and move himself/herself in it. In this way, the declarative competence of a space is built through the procedural competence, as we previously showed (Mandolesi et al., 2003). In the second case (vision from above/table RAM task), the participant is facilitated in the construction of the spatial cognitive map because he/she sees the maze in its completeness; therefore, his/her declarative knowledge is promptly formed. This interpretation would explain why WS individuals do not show procedural deficits in the free-choice paradigm of the table RAM task. In this line, it is intriguing to interpret the vision from above of the table RAM like an observation type that permits the observer to develop a sort of “perceptual blueprint” of the task to be learned (Bandura, 1977).

As last note, it is important to recall the functional role of dorsal stream in the spatial cognition (Ungerleider et al., 1998). fMRI studies showed that the dorsal stream projections to prefrontal, premotor, and medial temporal cortices through the posterior cingulate and retrosplenial cortices support spatial working memory, visually guided action, and navigation (Kravitz et al., 2011; Burles et al., 2018). Interestingly, in WS impairment of dorsal stream functionality, hypoplasia of the dorsal areas of the parietal cortex and weakening of fronto-parietal circuitry have been described (Bernardino et al., 2014). In the light of such an evidence, it is possible to hypothesize that in the resolution of the table RAM task a specific portion of the posterior cingulate cortex might be involved, linking thus the deficits of WS individuals to the vulnerability of the medial pattern of dorsal stream.

We are aware that a possible limitation of the present work concerns the relatively small sample size; however, it has to be considered that WS is a rare genetic disorder. In an attempt to obtain major consistency among performances, we carefully compared the WS individuals’ performances with those of mental age-matched TD children.

Presenting the table RAM task as a game, we allowed the study of the searching behavior in peripersonal space in an ecological manner. Furthermore, the table RAM is an easy task, particularly suitable for clinical populations with evident deficits. However, despite its simplicity, the table RAM task allows the evaluation of different facets of the spatial abilities.

In conclusion, the present study highlights that the difficulties in processing visuospatial information typically displayed by individuals with WS have to be extended also to the processing of visuospatial information of the peripersonal space. Further research on spatial impairment in WS will be carried by using RAM paradigms in virtual reality in a rehabilitative perspective.

## Data Availability Statement

The datasets generated for this study are available on request to the corresponding author.

## Ethics Statement

The studies involving human participants were reviewed and approved by the Ethics Committee of Bambino Gesù Children’s Hospital, Rome, Italy [protocol number: 486LB] and was carried out in accordance with the Declaration of Helsinki. Written informed consent was obtained from the minors’ legal guardian/next of kin for the publication of any potentially identifiable images or data included in this article.

## Author Contributions

All authors designed the research. SM and MP tested the participants. All authors analyzed the data and discussed the data. FF, PS, LP, and LM wrote the article.

## Conflict of Interest

The authors declare that the research was conducted in the absence of any commercial or financial relationships that could be construed as a potential conflict of interest.
